# Use of data mining techniques to investigate disease risk classification as a proxy for compromised biosecurity of cattle herds in Wales

**DOI:** 10.1186/1746-6148-4-24

**Published:** 2008-07-04

**Authors:** Ángel Ortiz-Pelaez, Dirk U Pfeiffer

**Affiliations:** 1 Epidemiology Division, Department of Veterinary Clinical Sciences, The Royal Veterinary College, University of London, Hawkshead Lane, North Mymms, Hatfield, Herts, AL9 7TA, UK

## Abstract

**Background:**

Biosecurity is at the forefront of the fight against infectious diseases in animal populations. Few research studies have attempted to identify and quantify the effectiveness of biosecurity against disease introduction or presence in cattle farms and, when done, they have relied on the collection of on-farm data. Data on environmental, animal movement, demographic/husbandry systems and density disease determinants can be collated without requiring additional specific on-farm data collection activities, since they have already been collected for some other purposes. The aim of this study was to classify cattle herds according to their risk of disease presence as a proxy for compromised biosecurity in the cattle population of Wales in 2004 for risk-based surveillance purposes.

**Results:**

Three data mining methods have been applied: logistic regression, classification trees and factor analysis. Using the cattle holding population in Wales, a holding was considered positive if at least bovine TB or one of the ten most frequently diagnosed infectious or transmissible non-notifiable diseases in England and Wales, according to the Veterinary Investigation Surveillance Report (VIDA) had been diagnosed in 2004. High-risk holdings can be described as open large cattle herds located in high-density cattle areas with frequent movements off to many locations within Wales. Additional risks are associated with the holding being a dairy enterprise and with a large farming area.

**Conclusion:**

This work has demonstrated the potential of mining various livestock-relevant databases to obtain generic criteria for individual cattle herd biosecurity risk classification. Despite the data and analytical constraints the described risk profiles are highly specific and present variable sensitivity depending on the model specifications. Risk profiling of farms provides a tool for designing targeted surveillance activities for endemic or emerging diseases, regardless of the prior amount of information available on biosecurity at farm level. As the delivery of practical evidence-based information and advice is one of the priorities of Defra's new Animal Health and Welfare Strategy (AHWS), data-driven models, derived from existing databases, need to be developed that can then be used to inform activities during outbreaks of endemic diseases and to help design surveillance activities.

## Background

Following stagnation during the late nineties, world trade in agricultural products has increased during the last six years [1] on average by 9% annually. This development together with the agreement of sanitary and phytosanitary measures (SPS agreement) for the protection of public, animal and plant health during international trade have resulted in an increased need for developing more reliable certification approaches of disease-free status at farm and national level. Farm assurance schemes and health plans have been established in European countries to allow farms to reach disease-free status, to improve farming and welfare standards and to ensure that safe animal products are offered to consumers.

The implementation of biosecurity and bio-containment has been a component of such approaches and both concepts have also been emphasized in other areas such as bioterrorism, contingency plans and animal disease emergency plans [2]. Biosecurity has been defined as 'a process to protect from attack or interference due to biological organisms' [3]. In the animal-farm context, biosecurity is a general concept that encompasses a variety of actions. It is subject to multiple approaches of different complexity and scope, ranging from protection against animal and wildlife contacts to vaccines and antibiotics. Official veterinary services in most countries regularly produce guidelines and instructions on biosecurity practices for farmers. In the United States the Bovine Alliance on Management and Nutrition (BAMN) was established to assist the cattle industry with management practices designed to control infectious diseases. BAMN publications are a detailed list of recommended biosecurity practices for cattle farmers [4]. The Department of Environment, Food and Rural Affairs (Defra) in the UK has developed several packages of "Biosecurity guidance to prevent the spread of animal diseases" [5].

These guidelines are usually a composite of common knowledge and evidence-based recommendations describing the Dos, Don'ts and rules of thumbs for every activity dealing with visitors, vehicles, animal management, feed and water, manure and manpower [6]. Instructions on how to design tailor-made biosecurity programmes using risk assessment approaches are also a common component of such information material.

Pinto and Urcelay [7] developed a biosecurity scoring system for pig farms based on 26 factors following the classification proposed by Barcelo and Marco [8]. The most important biosecurity factors are grouped into the location of the farm, the isolation of replacement stock and the farm and its different moveable, non-moveable, internal, health management procedures and animal welfare risk factors. Profiles of biosecurity measures based on purchase policy, transport and farm management and personnel practices in fattening swine herds were described using multivariate techniques in previous studies [9-11].

Few research studies have attempted to identify and quantify the effectiveness of biosecurity against multi-disease outcomes in cattle farms. When done, they have relied on the collection of data about on-farm biosecurity practices on a limited number of farms searching for associations between certain commonly used husbandry practices and the presence and/or introduction of individual infectious diseases. The challenge of obtaining representative epidemiological data becomes more relevant for surveillance and disease control programmes at the national level where policy makers must take into account the animal population as a whole [12]. Factors such as 'cattle removed from the farm for sale were allowed to return when not sold', 'grazed cattle at other farms' and 'the veterinarian always wears protective clothing' are biosecurity practices that allow direct/indirect animal contacts and were found to be significantly associated with the risk of introduction of bovine herpesvirus type 1 (BHV1), bovine viral diarrhoea virus (BVDV), Salmonella spp. and leptospirosis in dairy farms [13]. 'Cattle movement between farms' is a risk factor for Johne's disease [14], 'lack of quarantine of imported cattle' and 'communal grazing' are risk factors for disease introduction to beef-cow calf farms [15] and 'purchase of cattle', 'participation in cattle shows', and 'employee also working at other farms' are significant risk factors for the existence of BHV1 antibodies [16].

From an epidemiological perspective, disease occurrence in farm animal populations is determined by a multi-factorial causal web. The different factors, measurable or not, that increase or decrease the risk of introduction or presence of disease, act concurrently in space and time. Data on some disease determinants can be collated without requiring additional specific on-farm data collection activities, since it has already been collected for some other purpose. Determinants at the individual animal level are more difficult to obtain than those at the farm level, except where animal identification systems are in place. They can be divided into four categories: environmental, animal movements, demographic/farming-system patterns and densities. Intensive fattening pig units/areas which were very common in the 1970s and 80s are an example of where disease presence was very much determined by the combination of these four groups of factors.

The aim of this study was to classify cattle herds according to their risk of disease presence as a proxy for compromised biosecurity in the cattle population of Wales based on readily available data not requiring on-farm data collection, as part of a risk assessment leading towards more effective biosecurity risk management on cattle farms.

## Results

**Figure 1:**
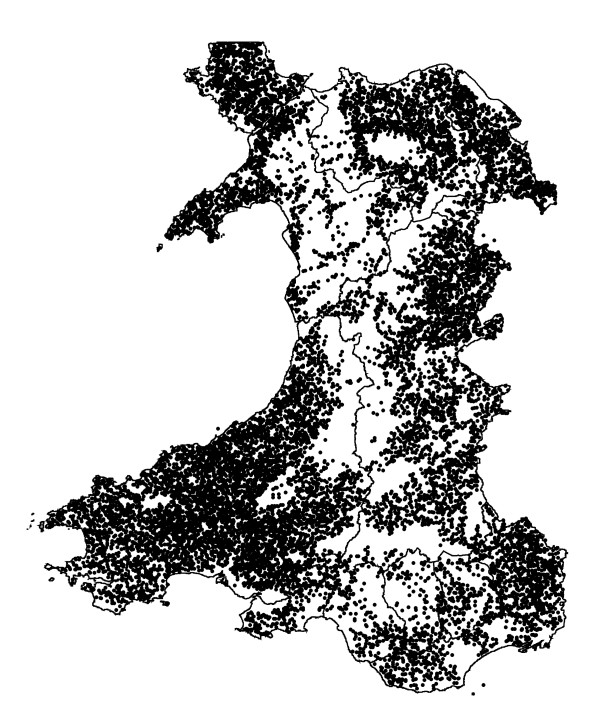
Location of the holdings in the study population.

The identification of the cattle holdings to be included in the study population was performed using a combination of three data sources: CPHs that submitted bovine samples or specimens to any VLA-Regional Laboratories during 2004 (2,721), CPHs that were tested for TB in Wales during 2004 (2,098) and CPHs that registered cattle movements into CTS during 2004 (13,352 registered movements off and 10,258 registered movements on). Twenty five CPHs which were located in neighbouring English counties but generally close to the Welsh border were included in the final study population. Non-farming holdings were excluded from the final study population. These included markets, abattoirs, show grounds, artificial insemination centres and collection centres. Thus it is expected that the number of included holdings which did not have a cattle farm enterprise was reduced to a minimum. The final study population included 15,845 CPHs which represents 110% of the 13,966 holdings reported by the Agricultural Census to have had cattle in 2004 [17]. The point locations of all cattle holdings in the study population are shown in Figure 1. The number of holdings that had been reported to have at least one of the listed diseases during 2004 was 1,372 (8.65%).

Four variables (Table 1) had more than 30% missing values: FARMA (47.1%), ADDPOST (33.2%), POPCAS (70.2%) and TVETNET (70.9%). The MCAR test was significant at 0.05 level for the four variables hence data cannot be assumed to be missing completely at random and these variables would introduce bias if included in the multivariable logistic regression model. The final random effect logistic regression model was based on data from 10,036 observations and a disease prevalence of 7.64%. The parish, included as a random effect (n = 889), had an intra-cluster correlation of 0.05 (95% CI: 0.046–0.068), indicating a very low, but significant, level of spatial dependence in the data.

**Table 1:** Description of the independent variables included in the analysis

**Name**	**Description**	**Source**	**Type**
**Demographics**			

**TVETNET**	Holding type. Reclassified into three categories: beef, dairy, other (including mixed herds).	VETNET. Animal Health Information System of the State Veterinary Service (SVS)	Categorical
**CATSHE**	Mixed holding (cattle and sheep).	Agricultural Census 2004	Binary
**TCTS**	Holding type. Only two types of holdings appear in the final study population: agricultural holdings with land and landless keeper. Other holding types as in CTS database do not appear because markets, abattoirs, Artificial Insemination Centres and show grounds have not been included in the study population.	Cattle Tracing System (CTS)	Binary.
**FARMA**	Total area farmed in Ha.	Agricultural Census 2004	Numeric
**OPENCLOSED**	Registered movements on/off in CTS during 2004. If none was recorded, it was considered closed. If at least one movement either on or off was registered, it was considered open.	CTS	Binary
**HERSIZE**	Herd size was estimated by combining the information available in two data sources. It is the number of cattle present at the time of conducting the Agricultural Census or when VETNET database was updated	VETNET and Agricultural Census (2004)	Numeric

**Densities**			

**CATDENSITY**	Average number of cattle in the area surrounding the holding's location. The kriged surface using HERSIZE is a "smoothed" value of the cattle population in the area where the holding is located. This is obtained by generating a cattle population density surface from interpolation of herd size numbers between the holding point locations. It was calculated by using ordinary kriging based on a spherical semivariogram using ArcGIS 9 (^© ^ESRI).	HERSIZE	Numeric
**SHEDENCEN**	Density of sheep in the area where the holding is located. This is calculated by assigning to each location the total sheep population of sheep in the 5 km^2 ^grid cell where the holding is located.	UKBORDERS-EDINA-Agricultural Census 2004	Numeric
**CATBUF5**	Density of cattle holdings in the buffer area of 5 km radius (78.5 km^2^) surrounding the point location of each holding of the study population.	Agricultural Census 2004, CTS and Farmfile	Numeric

**Environmental**			

**ADDPOST**	Number of addresses in the post code where the holding is located.	UK Postcode Directory-National Statistics	Numeric
**POPCAS**	Number of total human population in the Census Area Statistics (CAS) where the holding is located.	UK Census 2001-National Statistics. 2001 Census Aggregate Outputs. Economic & Social research Council (ESRC).	Numeric
**SOILCAT**	24 soil classes according to NATMAP soilscapes have been aggregated into four main soil types: Combinations of loamy soils, combination of acid soils, combination of freely draining soils and other types (peat soils, salt marsh, sand dune, etc.).	NATMAP soilscapes for Wales. National Soil Resources Institute (NSRI) – Cranfield University. Silsoe. Bedford.	Categorical
**TEXTCAT**	Three classes of soil texture according to NATMAP soilscapes: loamy, peaty and sandy.	NATMAP soilscapes for Wales. National Soil Resources Institute (NSRI) – Cranfield University. Silsoe. Bedford.	Categorical
**DRAINCAT**	Six classes of soil drainage according to NATMAP soilscapes and re-classified into four types: freely draining, impeded and slightly impeded drainage, surface wetness/naturally wet and variable.	NATMAP soilscapes for Wales. National Soil Resources Institute (NSRI) – Cranfield University. Silsoe. Bedford.	Categorical
**FERTCAT**	Ten classes of soil fertility according to NATMAP soilscapes re-classified into four types: high, moderate, low and lime-rich.	NATMAP soilscapes for Wales. National Soil Resources Institute (NSRI) – Cranfield University. Silsoe. Bedford.	Categorical
**LANDNATMAP**	Sixteen land classes according to LANDNATMAP re-classified into three types: combinations of arable land, combinations of grassland and other (moorland, forestry, etc.)	NATMAP soilscapes for Wales. National Soil Resources Institute (NSRI) – Cranfield University. Silsoe. Bedford.	Categorical.
**LANDCOVER**	Ten classes: improved grassland, coniferous woodland, semi-natural grass, broad-leaved/mixed woodland, mountain/heath/bog, arable and horticultural, built-up/gardens, standing open water, oceanic and coastal. Source: 1 km grid raster data with aggregate classes and sub-classes,	Land Cover Map 2000: Centre for Ecology & Hydrology.	Categorical
**HABICAT**	Twenty classes of habitats according to NATMAP soilscapes and re-classified into four types: combinations of pasture and woodlands, combination of grassland and grass moors, combination of wet areas and other (coastal salt marsh, sand dune vegetation, etc.).	NATMAP soilscapes for Wales. National Soil Resources Institute (NSRI) – Cranfield University. Silsoe. Bedford.	Categorical
**TOTRAIN**	Total rainfall in 2003. 5 km grid monthly mean rainfall	Mean rainfall and temperature data (1999–2004). Geographic Information Unit- DEFRA- authorised by the Met Office	Numeric
**AVETEMP**	Annual average surface temperature in 2003. 5 Km grid monthly mean temperature	Mean rainfall and temperature data (1999–2004). Geographic Information Unit- DEFRA- authorised by the Met Office	Numeric
**5KMAONB**	Within or at less than 5 Km to an Area of Outstanding Natural Beauty (AONB).	Protected sites datasets Country side Council of Wales	Binary
**5KMNNR**	Within or at less than 5 Km to a National Nature Reserve (NNR).	Protected sites datasets Country side Council of Wales	Binary
**5KMLNR**	Within or at less than 5 Km to a Local Nature Reserve (LNR).	Protected sites datasets Country side Council of Wales	Binary
**WITHISSSI**	Within a "Site of Special Scientific interest" (SSSI).	Protected sites datasets Country side Council of Wales	Binary

The fixed effects included in the final model were: cattle density, herd size, total annual rainfall, average temperature, within or at less than 5 km of an AONB and open/closed herd (Table 2). High local cattle density, large herd size and being open are associated with a significant increase in the odds of having any of the selected diseases. Increased rainfall, higher average temperature and being within or at less than 5 km of an AONB reduce the odds for the presence of diseases in the study population. Both categorical variables TOTRAIN and FARMA have a linear association with the outcome of interest. Classification ability of the model for different cut-off points is displayed in Table 3.

**Table 2:** Results of the multivariable logistic regression models

	Model 1			Model 2			Model 3		
	
**Variable names**	**OR**	**P value**	**95% CI**	**OR**	**P value**	**95% CI**	**OR**	**P value**	**95% CI**
Average number of cattle in the area surrounding the holding's location (CATDENSITY (binary) > 2630	2.46	P < 0.001	1.84–3.3	2.62	P < 0.001	1.88–3.64	2.59	P < 0.001	1.92–3.5
Herd size (HERDSIZE) (categorical) < 30									
30–88	3.86	P < 0.001	2.81–3	3.22	P < 0.001	2.23–4.64	3.77	P < 0.001	2.75–5.2
> 88	10.34	P < 0.001	7.65–11	6.62	P < 0.001	4.55–9.63	9.4	P < 0.001	6.9–12.8
Total farmed area (FARMA) (categorical) < = 40Ha				1.26*	P = 0.001	1.09–1.45			
41–100Ha							1.15	P = 0.183	0.93–1.43
> 100Ha							1.31	P = 0.007	1.07–1.6
Within or at less than 5 Km to an Area of Outstanding Natural Beauty (5KMAONB) (binary) Yes	0.69	P = 0.023	0.5–0.95	0.63	P = 0.01	0.44–0.7	0.7	P-0.027	0.5–0.96
Total rainfall in 2003 (TOTRAIN)	0.77	P < 0.001	0.68–0.88	0.77	P < 0.001	0.67–0.87	0.76		0.67–0.87
Annual average surface temperature in 2003 (AVETEMP) (categorical) < 9°C	0.81*	P = 0.003	0.7–0.93						
> = 9–11°C				0.88	P = 0.299	0.77–1.11	0.8	P = 0.043	0.64–0.99
> = 11°C				0.72	P = 0.044	0.53–0.99	0.68	P = 0.007	0.51–0.9
Registered movements on/off in CTS during 2004 (OPENCLOSED) (binary) Yes	6.08	P < 0.001	2.98–12.4	2.18	P = 0.095	0.87–5.44	6.2	P < 0.001	3–12.74

Model 1: No. observations: 10,036. Variables with less 30% missing values

Model 2: No. observations: 7,412. Model 1 plus FARMA. No imputation.

Model 3: No. observations: 10,036 Model 1 plus FARMA with imputed missing values (mean)

OR based on linear association

**Table 3:** Classification performance of the three classification trees using different misclassification costs and the logistic regression models using different cut-off points

Classification trees	Misclassification costs			
	Tree 1:1	Tree 5:1	Tree 10:1	Tree 20:1

Sensitivity	0%	16%	64.3%	91%
PPV	0%	23.2%	15%.	11.9%
Specificity	100%	95%	65.5%	36.1%
NPV	91.3%	65.8%	95%	97.7%
Error rate	8.7%	11.9%	34.6%	59.1%
AUC		71%	71%	67.6%
				

Logistic regression	Classification cut-offs			

	0.5	0.3	0.2	0.1
Sensitivity	0%	1.1%	10.8%	65.5%
PPV	0%	22.3%	27.6%	15.5%
Specificity	100%	99.7%	97.4%	67.3%
NPV	91.64%	91.7%	92.3%	95.5%
Error rate	8.4%	8.6%	9.8%	32.8%
AUC	72%	72%	72%	72%

Out of the four tested variables with missing values, the one with the lowest P-value was used to assess the impact of its inclusion in the final model. Two models were fitted adding to the above-reported one (Model 1) the variable FARMA with missing values (Model 2) and FARMA with imputation of missing values using the three imputation methods (Model 3: with mean imputation is shown in Table 2). These two models include FARMA as significantly associated with the outcome once adjusted for the other variables. Results of the three models are shown in Table 2.

**Figure 2:**
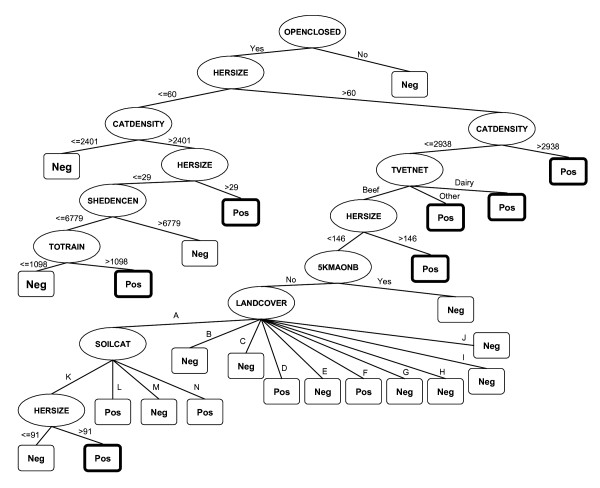
**Classification tree 10:1**. Oval branch nodes = splitting variables. Rectangular terminal nodes include classification as positive (Pos) or negative (Neg). Terminal nodes classified as positive and with more than 200 observations are framed in bold. Legend: A: improved grassland. B: coniferous woodland. C: semi-natural grass. D: broad-leaved/mixed woodland. E: mountain/heath/bog. F: arable and horticultural. G: built-up/gardens. H: standing open water. I: oceanic. J: coastal. K: combinations of freely draining foodplan soils. L: fen-peat soils. M: combination of acid and loamy soils. N: other types (salt marsh, sand dune, etc.).

All variables included in the classification trees are shown in Table 4 in hierarchical order where variables at the top of the table have the highest discriminatory ability and therefore appear at the top of the classification trees. Tree 1:1 is unable to split the data with the available variables hence all observations are classified as negative, the most prevalent category of the outcome. Classification performance of each of the trees is shown in Table 3. As an illustration the tree 10:1 is displayed in Figure 2.

**Table 4:** Hierarchical ranking of the variables in the four classification trees

**Tree 1:1**	**Tree 5:1**	**Tree 10:1**	**Tree 20:1**
	OPENCLOSED	OPENCLOSED	OPENCLOSED
	HERSIZE	HERSIZE	HERSIZE
	CATDENSITY	CATDENSITY	CATDENSITY
	TVETNET	TVETNET	FARMA
	CATSHE	5KMAONB	LANDCOVER
	CATBUF5	LANDCOVER	TOTRAIN
		SHEDENCEN	
		SOILECAT	

The classification or decision rules extracted from terminal nodes classified as positive in the three trees are described below with the number of observations and percentage of false positives in brackets:

Tree 5:1:

OPEN, HERDSIZE > 60 and CATDENSITY > 2938 (1227, 35.8%)

OPEN, HERDSIZE > 193, CATDENSITY < 2938 and DAIRY (845, 44.9%)

OPEN, HERDSIZE > 190, CATDENSITY < 2938, BEEF, with SHEEP and CATBUF5 < 70 (336, 45.4%)

Tree 10:1:

OPEN, HERDSIZE > 60 and CATDENSITY > 2938 (1612, 22.1%)

OPEN, HERDSIZE > 60, CATDENSITY < 2938 and DAIRY (3564, 39.6%) or OTHER (272, 46.4%)

OPEN, HERDSIZE > 146, CATDENSITY < 2938 and BEEF (1521, 38%)

OPEN, HERDSIZE 29–60, CATDENSITY > 2401 (628, 38.7%)

OPEN, HERDSIZE 91–146, CATDENSITY < = 2938, BEEF, 5KMAONB (No), "Improved grassland" (LANDCOVER) and "Freely draining floodplain soils" (SOILCAT) (278, 41.8%)

OPEN, HERDSIZE < = 29, CATDENSITY > 2401, SHEDENCEN < = 6779 and TOTRAIN > 1098 mm (558, 38.6%)

Tree 20:1:

OPEN, HERDSIZE > 51 (10934, 25.11%)

OPEN, HERDSIZE < 51 and CATDENSITY > 2479 (988, 30.72%)

OPEN, HERDSIZE 43–51, CATDENSITY < 2479, FARMA > 44, "Improved grassland" (LANDCOVER) (295, 37.4%)

OPEN, HERDSIZE < = 43, CATDENSITY < 2479, FARMA > 44, "Improved grassland" (LANDCOVER) and TOTRAIN < = 967 (490, 37.53%)

Only tree 10:1 has a balanced error rate with a sensitivity and specificity of 64 and 65%, respectively. Tree 5:1 has low sensitivity and high specificity. At the other extreme, tree 20:1 improves the proportion of correctly classified positive observations substantially at the expense of a lower specificity and the highest overall error rate. Positive predictive values remain low in the three trees with a high proportion of false positives in trees 10:1 and 20:1. The range of false positives generated by individual rules classified as positive varies between 25 and 46.6%. Amongst the rules for defining positive holdings, tree 10:1 has the lowest percentage of misclassified observations (22.1%) for a rule involving the variables OPENCLOSED and HERDSIZE and CATDENSITY, followed by a rule in tree 20:1 with 25.1% of positives being false positives, based on the variables OPENCLOSED and HERDSIZE.

**Figure 3:**
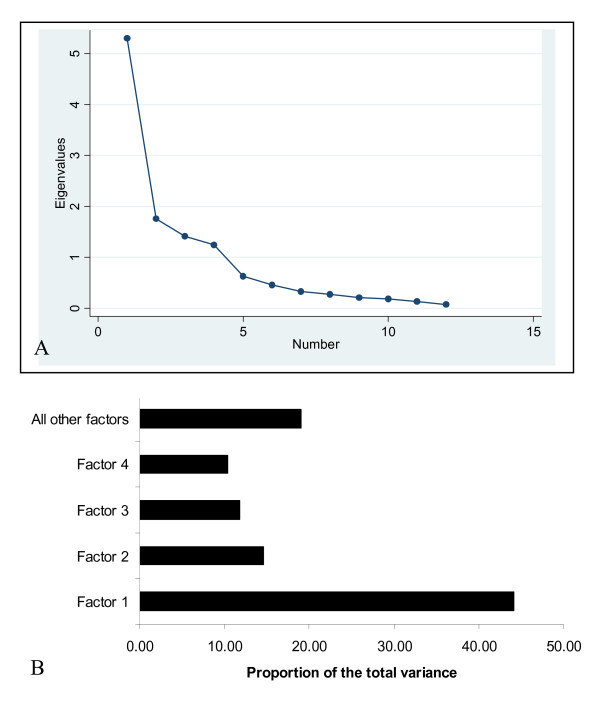
A: Initial eigenvalues of the factors. B: Variability explained by the factors.

The 66 correlation coefficients between all movement variables were significant at 0.05% level using the Bonferroni adjustment. The KMO index of sample adequacy of 0.75 supports the use of factor analysis. Four factors have eigenvalues greater than 1 (Figure 3a), accounting for 81% of the total variance of the dataset (Figure 3b and Table 5). Factor loadings of the rotated solution are displayed in Table 6.

**Table 5:** Output of the factor analysis

**Factors**	**Initial Eigenvalues**		
	
	**Total**	**% of Variance**	**% Cumulative**
1	5.3	44.18	44.18
2	1.7	14.63	58.82
3	1.4	11.78	70.6
4	1.2	10.37	80.98
5	0.6	5.21	86.19
6	0.4	3.80	89.99
7	0.3	2.74	92.74
8	0.27	2.29	95.03
9	0.2	1.73	96.77
10	0.18	1.53	98.3
11	0.13	1.09	99.4
12	0.07	.6	100.00

Eigenvalues, proportion of variance and cumulative proportion of variance for the 10 main factors extracted from the factor analysis of the twelve movement variables of Welsh cattle holdings in 2004

**Table 6:** Rotated Component Matrix of final factors loadings for a factor analysis of the twelve movement variables of Welsh cattle holdings in 2004 (loadings > 0.5 in bold)

	Factor			
	
	1	2	3	4
ANITOOUTWALES	**0.550**	0.162	**0.684**	-0.128
LOCTOOUTWALES	0.046	0.190	**0.778**	0.363
DAYTOOUTWALES	0.255	0.199	**0.831**	-0.014
ANIONOUTWALES	0.391	**0.812**	0.003	-0.002
LOCONOUTWALES	-0.017	**0.803**	0.273	0.211
DAYONOUTWALES	0.128	**0.891**	0.217	0.029
ANITOWITHINWALES	**0.681**	0.287	-0.155	**0.500**
LOCTOWITHINWALES	0.101	0.062	0.231	**0.872**
DAYTOWITHINWALES	0.325	0.073	-0.040	**0.811**
ANIONWITHINWALES	**0.923**	0.158	0.178	0.082
LOCONWITHINWALES	**0.772**	0.136	0.288	0.263
DAYONWITHINWALES	**0.825**	0.077	0.260	0.212

The first factor accounts for 44% of the variance and has strong positive loadings (> 0.5) for ANITOOUTWALES ANITOWITHINWALES, ANIONWITHINWALES LOCONWITHINWALES and DAYONWITHINWALES, and can be labelled as the most frequent pattern, with movements off to within and outside Wales and movements on within Wales. The second factor accounts for 14.6% of the total variance and is highly correlated with ANIONOUTWALES, LOCONOUTWALES, and DAYONOUTWALES labelled as 'animal movements from outside Wales'. The third factor accounts for 11.8% of the variance and has strong positive loadings for ANITOOUTWALES, LOCTOOUTWALES, and DAYTOOUTWALES and represents 'frequent off movements to outside Wales'. The last factor accounts for 10.4% of the total variance and has strong positive loadings for ANITOWITHINWALES, LOCTOWITHINWALES, and DAYTOWITHINWALES. It therefore represents 'frequent off movements to within Wales'.

When included in a multivariable logistic regression analysis, factors 3 and 4 are linearly associated with Odds Ratios 1.14 (95% CI: 1.09–1.2 P < 0.001), and 1.65 (95% CI: 1.57–1.72 P < 0.001), respectively. The other two factors were not statistically significant.

## Discussion

This study has pursued an epidemiological approach towards the investigation of biosecurity, as a broad concept, and disease presence where the three constitutive elements of the livestock farming systems, namely, human community, animal population and biophysical environment [18], are assumed to have an important influence on the health status of an agricultural holding. Various assumptions in relation to the data need to be discussed here prior to interpreting the results. Biosecurity and environmental attributes are linked to holdings based on the point location of the geo-references. This may lead to misclassification, particularly for large farms with multiple parcels of land. This will also be the case for multi-herd farm units under the same CPH but applying different husbandry systems and, in certain occasions, with permanent physical separation. It has been suggested that such positional error of geo-coded addresses in epidemiological studies introduces systematic errors into the analysis [19]. The collation of data from multiple sources results in a large number of variables, non-linear dependency structures, missing values, imprecise data and errors [20]. The benefit of merging data sets with different types of information about the same holding for providing evidence of the association between disease and risk factors has been acknowledged [21]. As highlighted by James [12], sources of data on animal identification, registration, movement, health and production are frequently incomplete and in incompatible formats. The databases explored in this study have very different characteristics, particularly in relation to objectives; time periods covered and database structure. Some store data about animals (Farmfile, CTS), and others about the holding (Agricultural Census). Some holding data are imputed and therefore may not reflect the true value for a particular farm (Agricultural Census). Several databases are not updated on a yearly basis (UK Census, EDINA and VETNET).

Surveillance data often include missing values that need to be assessed prior to any analysis. In the case of the logistic regression it has been shown that the analysis of patterns of missing values is necessary to avoid complete case analysis where only observations without missing values are included in the final model. If the missing-data pattern is not MCAR, it can lead to loss of precision and bias in the analysis results [22]. The variable FARMA with mean-imputed values replacing missing values was significantly associated with the outcome, and its inclusion in the model resulted in slight modification of the effect estimates for CATDENSITY, HERSIZE, AVETEMP and OPENCLOSED (see Table 3). However, mean imputation is not a preferred method for missing value imputation since the resulting reduction in variance can lead to bias in estimates of other or all variables in the regression analysis [23]. In this regard when other simple imputation methods were applied (regression and mean of nearby points) the new variable FARMA was not included in the final model.

The risk factors included in the analysis describe holding rather than individual animal characteristics. The outcome of interest was whether at least one from a group of diseases had been diagnosed on a holding since the goal of this study was the generic disease risk classification of holdings based on factors not collected on farm. It was assumed that the variables considered in this analysis are potential risk factors for all selected diseases and that the risk factors equally affect the presence of each individual disease. This assumption is partially violated for various reasons. Firstly with such an aggregated outcome, the variation in prevalence between diseases may influence the relative weight of each disease in the analysis, and thereby introduce bias towards the ability of being more likely to detect the more common disease's risk factors. The pool of diseases used to define the outcome also has the effect of increasing the baseline exposure of the population to pathogens due to higher prevalence compared to the analysis at individual disease level. The criterion used to define the outcome variable does not consider the number of different diseases diagnosed in the study units, but instead if at least one of the selected ones had been reported. Amongst positive holdings (1,372), BTB is the most frequent disease (46.4%) followed by mastitis (25.7%) and rotavirus (8.6%) Sixteen percent of the positive holdings had been diagnosed with multiple diseases: two holdings were diagnosed to have four diseases, 34 had 3 diseases confirmed and 190 had confirmation of two.

The imbalance in the prevalence of the diseases included in the outcome could have led to a potential bias of the risk factor identification towards BTB. To address this issue- the three logistic regression models were re-fitted giving double weight to observations positive to 'any other disease' but negative to BTB by adding to the dataset the 666 observations that met this criterion. The new dataset contained 16,511 observations, 2,038 of the positive (1,372 positive in the initial dataset + 666 positive to any of the other diseases). The prevalence was now 12.34% compared to 8.65%, and the proportion of holdings positive to BTB was reduced from 46.4% to 31.25% in the new dataset. The only variable dropped from the model without variables with missing values over 30% (Model 2) was open/closed (OR = 1.8 P = 0.115. 95% CI: 0.86–3.73). This finding indicates that this variable is the most influential for the presence of BTB and that the other variables can be considered generic risk factors for the presence of disease.

Secondly it is likely that the Farmfile disease data introduced an unknown degree of selection bias given that the VLA scanning surveillance system relies on the voluntary submission of samples or specimens usually by private veterinary practitioners. This fact may have introduced an unknown proportion of false negative holdings if diagnoses were performed by private sources, unaccounted for in this study. However the diagnostic services of the VLA are the ones most frequently used by cattle farmers in Wales (personal communication). In contrast, bovine BTB data tends to be subject to a geographical bias as a result of variation in testing frequency. Yearly testing, for example, occurs predominantly in South Wales, and BTB is likely to be more affected by the movement pattern [24]. The inclusion of mastitis as one of the diseases defining the outcome variable is likely to have introduced a differential detection bias, as dairy herds are subject to regular serological monitoring via bulk milk, whereas beef herds are not. As it could not be determined from this data whether any reported disease occurred endemically within a particular herd or had been newly introduced, the analysis had to be about identifying risk factors for the presence of disease during that particular period.

Despite these two sources of bias, inherent to many epidemiological analysis of this kind, the approach provides a generic measure of the overall risk of disease for the different farm profiles. Data collection based on longitudinal studies allowing more accurate ascertainment of disease status would significantly strengthen investigations aimed at quantifying the impact of biosecurity factors on the risk of disease introduction. The comparison of the results of more targeted studies with the ones reported in the current study would be inappropriate. The described high-risk profiles could be considered as the attributes of cattle holdings in Wales where the overall health status was compromised due to the increased likelihood of having diseases which are commonly present in the cattle population of the UK.

By reducing the cut-off points of the predicted probability in the logistic regression the sensitivity of the model increases at the expense of the specificity in similar fashion to the misclassification penalty in the classification trees. When introducing differential misclassification costs in the classification tree method the resulting tree may include other variables, whereas the reduction of the cut-off point in the logistic regression will not be associated with a different set of variables included in the final model. In terms of predictive ability both methods perform very similar, as reflected in AUC of 72% in the logistic regression and 71% in the classification trees, finding that has also been reported in other studies comparing these methods [25,26]. In the current study the misclassification penalties and the shift of the cut-off points were applied following the criterion that targeted surveillance should yield both higher sensitivity and higher positive predictive value than surveillance conducted randomly across a population [27]. If an economic factor such as the payment of compensation for finding disease has to be taken into account then the consequences resulting from misclassification of negative holdings, i.e., the specificity, would be an issue to consider.

The biological and/or epidemiologically plausible explanation of some of the classification criteria in the models can be difficult. Epidemiological studies are usually focussed on obtaining a good understanding of the explanatory variables included in the analysis, their interactions and quantifying the effect on the outcome [28]. The combination of the outputs of the analytical methods applied here provided a deeper insight into the structure of the data and the relationships between explanatory variables and the outcome of interest. In this respect, logistic regression had the advantage of giving a measure of the strength of the association between individual risk factors and the outcome, the classification trees included different sets of variables depending on the specified misclassification costs and factor analysis captured the essential aspects of movement patterns from a set of highly correlated variables.

Although the use of the latent score variables generated by the factor analysis results in the loss of the original movement variables, the factors together are able to represent the main characteristics of the movement pattern of Welsh cattle herds. The loss of individual directly measurable variables is compensated for by the strength of the technique in generating multiple less collinear independent variables which should still allow biological interpretation [28,10]. In this case, four movement patterns factor score variables account for most of the variability within and between the 12 original movement variables. The four factors selected in this study indicate that holdings in Wales can be split in terms of movement patterns into two main groups: standard pattern of animal movements outside and within Wales represented by factors 1 and 2, together with a more extreme pattern of those moving mainly animals off their premises to outside and within Wales, represented by factors 3 and 4. The linear association between scores and the outcome of interest has to be interpreted with caution given that the scores for each factor have been scaled to have mean 0 and variance 1. Therefore, the strength of the association is not meaningful and only whether the factor increases or decreases the likelihood of the outcome is worth looking at. The association of the individual scores of factors 3 and 4 with the outcome reveals the risk associated with the frequency of off movements and the number of different locations to which animals are moved to rather than the total number of animals moved. In contrast, factors 1 and 2, which account for more than half of the total variance and represent a more standard movement pattern, are not positively associated. In terms of country movements, factors 3 can be also be labelled as movements to holdings outside Wales and factor 4 with off movements to holdings within rather than outside Wales. This pattern may indicate that introduction of infectious diseases via fomites (equipment, vehicles) may be more important than the introduction of infected/diseased animals. Although speculative, a possible explanation for this could be that holdings trading locally would tend to use their own means of transport. In contrast, movements to locations outside Wales may involve external professional haulage, who may be more aware and compliant with good biosecurity practices.

Despite the data and above-mentioned methodological constraints, the combined results of the analysis showed a distinctive high-risk profile for having the multiple-disease outcome characterised by open large cattle herds located in high-density cattle areas with frequent movements off to many locations within Wales. Additional risks are associated with the holding being a dairy enterprise and farming a large area. Decreased risk is linked to the holding being located in areas with high rainfall and mild temperature or close to or within "areas of outstanding natural beauty" (AONBs). The latter is similar to national parks without the specific purpose of recreation and "consistent with the needs of agriculture, forestry and other uses" [29]. Farmers and landowners in AONBs are encouraged to conserve and enhance the landscape and discourage the draining and ploughing up of pasture [30]. They are remote areas with significantly less human population (t-test of ADDPOST: t = 6.5. P < 0.001) and cattle population (t-test of CATDENCEN: t = 9.26 P < 0.001) hence they may be areas of lower direct/indirect exposure to pathogens. The biological link of some of the risk factors such as rainfall and average temperature with the outcome variable will be indirect, acting through other intermediate pathways. For example, rainfall and temperature may determine the types of surfaces and bedding, grazing patterns, vegetation, infiltration capacities and grass cover, which might affect the persistence and spread of infectious agents and increase their likelihood of contact with cattle.

The positive decision rules extracted from the trees allow a more detailed description of the risk profiles. For instance in tree 10:1, non-beef herds that are open, large (> 60) and in lower cattle density areas (< 2938) are classified as positive, of which 40% are false positives. For a beef herd in the same area to be included in the high-risk group, it has to be larger (> 146) and the resulting false positives are 46%. If the beef herd is not quite as large (91–146) and located in a lower cattle density area (< 2938), tree 10:1 requires the addition of three environmental factors, TOTRAIN, SOILCAT and LANDCOVER, to be classified as positive. If the sensitivity of the risk profiles is to be increased (tree 20:1), open smaller herds (< 50) in lower density areas (< 2479) require a larger farmed grassland area (> 44 Ha) to be classified as positive with the consequent increase in the number of false positives from 25 to 37%.

It has been shown in this study that the combination of the movement pattern, herd size, local cattle density, herd type and farmed area are the major drivers in the definition of risk profiles for disease presence in cattle herds in Wales in 2004. This study has explored an alternative methodology for the definition of high-risk farm profiles based on factors not collected on farm as proxy measures of their biosecurity status as far as the risk of disease presence is concerned and not as the farming practices applied by the farmers. Alternatively the description of low-risk profiles could have been addressed using the outputs of this study and inform on holdings of low priority for interventions. The classification of the factors into four distinctive groups has been useful when interpreting the results of the analysis. The integration of area-level factors (environmental and densities) with holding-level factors (demographics and movements) in the risk profile prevents the analysis from producing a purely spatial predictive model whose main outcome would be the classification of areas at higher risk in Wales. Demographic and movement characteristics of a particular holding may increase its risk despite being located in an area of low risk due to its environmental attributes. This holistic approach towards biosecurity and risk of disease presence does not deny the importance of conducting further studies where purely spatial prediction models or specific risk profiles for particular diseases are developed.

Generic biosecurity is probably one of the areas where policy and advice have been influenced primarily by knowledge and experience. The combination of this type of expertise with research findings obtained from data-driven approaches should result in a better informed decision-making process. The need to provide new and robust evidence for biosecurity strategies has been recognised in many countries [31,32].

The development of generic risk profiles could help policy makers prioritize their actions in promoting preventative measures among high-risk holdings as well as in the case of localised outbreaks of endemic diseases or the design of surveillance programmes. The definition of sub-populations that are expected to have higher prevalence of disease is an essential component of the design of risk-based surveillance [27]. Further environmental and social variables could be considered with these types of models to enhance the classification criteria according to their risk of disease introduction/presence. Risk profiles can also be externally validated and used as part of on-farm risk assessment for informing the design of cost-effective farm-specific biosecurity risk management strategies.

## Conclusion

This work has been a first attempt at mining various livestock-relevant databases to obtain generic criteria for individual cattle herd biosecurity risk classification. The approach has demonstrated its potential, and should be used as one of many other reasons for working towards enhanced data quality, with more accurate disease status definition being a priority area. But even the outputs generated in the current study already can be used for informing risk-based surveillance activities on the basis of individual farm's risk profiles. It needs to be communicated though that the specified risk profiles are highly specific and present variable sensitivity depending on the model specifications. Although the analytical results are specific to the cattle population in Wales, similar studies can be conducted on other populations in the UK or elsewhere to obtain geographically tailored risk profiles. As the delivery of practical evidence-based information and advice is one of the priorities of Defra's new Animal Health and Welfare Strategy (AHWS) [31], data-driven models, derived from existing databases, need to be developed that can then be used to inform activities during outbreaks of endemic diseases and to help design surveillance activities.

## Methods

### Data

Cattle holdings were identified using the unique CPH (County-parish-holding) number for which a geo-reference could also be obtained. The location of each holding was based on the easting and northing coordinates obtained from the Agricultural Census database. The county number used by the CPH nomenclature corresponds to the distribution of county names prior to 1994 whereby Wales was divided into 8 counties: Powys, Gwynedd, Dyfed, Clwyd, South Glamorgan, Mid Glamorgan, West Glamorgan and Gwent.

The State Veterinary Service's VETNET database was used to extract BTB testing data. Data on non-notifiable diseases was retrieved from the Veterinary Laboratories Agency's (VLA) FarmFile database for all holdings in Wales for 2004. From these reports, ten diseases were selected according to a two-fold criteria: they can be considered as proxies for compromised biosecurity and are the most frequently diagnosed infectious or transmissible non-notifiable diseases in England and Wales by the VLA, according to the Veterinary Investigation Surveillance Report (VIDA) 2004 [33], except cryptosporidiosis due to lack of data. A holding was considered positive if at least bovine TB (BTB) or one of the following selected diseases was confirmed in 2004: bovine viral diarrhoea (BVD) (395 diagnoses), mucosal disease (330), infectious bovine rhinotracheitis/infectious pustular vulvovaginitis (IBR/IPV) (215), Neospora caninum (279), Johne's disease (2,328), salmonellosis (S. typhimurium) (118), rotavirus infection (1,114), mastitis due to: *Streptococcus uberis, Staphilococcus aureus *coagulase positive, *E. coli*, *Streptococcus dysgalactiae, Actynomices piogenes, Streptococcus agalactiae*, organisms not otherwise specified (NOS), yeast/fungi, *Pseudomonas spp *and *Staphilococcus *not otherwise specified (NOS) (3,773); pneumonia (10 causative agents) (1,506), fasciolosis (633).

A total of 37 putative risk factors, for disease occurrence in cattle herds based on husbandry system, movement pattern, human and animal population characteristics and ecological features of the geographical point location of the holding, divided in two groups, were considered in this analysis. Twenty-five of these were extracted from various data sources and grouped as follows: 6 on demographics/farming system, 3 on human and animal densities and 16 on environmental factors. Definitions of the variables and data sources are presented in Table 1. All cattle movements recorded in the Cattle Tracing System (CTS) where the origin or destination of the movement was a CPH in Wales during 2004 were extracted. In order to identify actual movements on/off of live animals for every holding, births were removed as well as deaths either recorded at abattoirs or on farms. Another 12 movement variables as described in Table 7 were calculated for the study population.

**Table 7:** Description of variables describing cattle movement characteristics of Welsh holdings in 2004

**Variable**	**Description**
**ANITOOUT**	Total number of animals moved out to holdings outside Wales
**LOCTOOUT**	Total number of locations outside Wales where animals were moved to
**DAYTOOUT**	Total number of days of movements of animals to holdings outside Wales
**ANIONOUT**	Total number of animals moved on from holdings outside Wales
**LOCONOUT**	Total number of locations outside Wales where animals were moved from
**DAYONOUT**	Total number of days of movements of animals from holdings outside Wales
**ANITOWITHIN**	Total number of animals moved out to holdings within Wales
**LOCTOWITHIN**	Total number of locations within Wales where animals were moved to
**DAYTOWITHIN**	Total number of days of movements of animals to holdings within Wales
**ANIONWITHIN**	Total number of animals moved on from holdings within Wales
**LOCONWITHIN**	Total number of locations within Wales where animals were moved from
**DAYONWITHIN**	Total number of days of movements of animals to holdings within Wales

### Analytical methods

Three data mining techniques have been applied to the study dataset according to the following analytical strategy. A previous assessment of the missing values of the 25 non-movement variables was used to inform the number of variables to be included in the logistic regression model. In parallel the same set of variables were analysed using a classification tree algorithm in order to build classification rules. The 12 movement variables were independently analysed using factor analysis. Finally the outputs of the three methods applied were integrated in qualitative risk profiles described in the discussion. Variables with more than 30% of values missing were discarded in the initial analysis. Analysis of missing values in discarded variables was conducted using Little's missing completely at random (MCAR) test [22] available in SPSS for Windows Release 15.0 (Chicago SPSS Inc.). Factors associated with the presence of any of the selected diseases at holding level during 2004 were identified by fitting a multivariable logistic regression model with parish as a random effect. The criterion for inclusion was forward selection based on the likelihood ratio test using a cut-off p-value of 0.05 for entry of variables into a preliminary model. The Akaike Information Criterion (AIC) was applied to compare model fits using continuous and categorical scale versions of the same variables to assess linearity of effects, as well as tests for interaction. The log likelihood was estimated using the Gauss-Hermite quadrature approximation and stability of the estimates was tested by comparing using different numbers of quadrature points. The final model used 24 quadrature points and none of the coefficients varied more than 10^-4 ^(0.01%).

Classification ability of the model was estimated using different cut-off points of the probability of the outcome and the areas under the receiver-operating characteristic (ROC) curves (AUC). The impact of including variables with missing values was assessed by comparing models using data based on three missing value imputation methods; means, regression and mean of nearby points. The latter was implemented by using as the span of nearby points the five valid values above and below the missing value in a sorted dataset by CPH, which is a variation of the sequential hot deck ordered by a covariate imputation method as described by Little and Rubin [22].

Environmental attributes for each holding were extracted using ArcGIS 9 (^©^ESRI) from the relevant GIS layers, and attributed to the corresponding farm locations using the "join" function of the ArcView Spatial Analyst extension (^© ^ESRI). Numeric variables were transformed into tertile categories. Nominal environmental variables were aggregated into a subset of categories based on biological considerations.

The twenty-five variables were served to the classification tree algorithm C4.5 [34] implemented in the statistical software WEKA 3.4.4 (^©^1999–2005 University of Waikato, New Zealand). The trees are built using binary recursive partitioning which involves the dataset successively being split into increasingly homogeneous subsets according to a pre-specified criterion [35], in this case, the presence/absence of disease. Starting from the root node containing all observations each split allocates them into mutually exclusive subsets (decision nodes) until a node has no further splits, resulting in terminal nodes (see Figure 2). The objective at each split is to maximize the proportion of observations with one of the outcome categories in the resulting nodes. The variable used to split a node and the decision on when the partitioning of a node ends is done by using the information gain criterion which is based on the concept of entropy [36]. If an entity *S *of size s contains *s*_*i *_elements of class *Ci *for i = {1, ..., m}, the amount of information needed to decide if an arbitrary example in *S *belongs to *Ci *is defined as:

$$I(s_{1},...,s_{m})=-\sum_{i=1}^{m}\left(\frac{s_{i}}{s}\right)\log_{2}\left(\frac{s_{i}}{s}\right)$$

In a dataset of *p *positive observations and *q *negative observations out of *N*, the difference of entropy in the dataset before and after the split using a binary variable *i *with categories *A *and *B *where *x *positive out of *r *observations contain category *A *and *y *positive out of *z *observations contain category *B*, is the information gain and is calculated as follows:

$$I\left(p,q\right)=-\left(\frac{p}{p+q}\right)\times \log_{2}\left(\frac{p}{p+q}\right)-\left(\frac{q}{p+q}\right)\times \log_{2}\left(\frac{q}{p+q}\right)$$

$$Entropy\left(i\right)=\left(\frac{r}{N}\right)\times Entropy\left(A\right)+\left(\frac{z}{N}\right)\times Entropy\left(B\right)$$

Being

$$Entropy(A)=-\left(\frac{x}{r}\right)\times \log_{2}\left(\frac{x}{r}\right)-\left(\frac{r-x}{r}\right)\times \log_{2}\left(\frac{r-x}{r}\right)$$

$$Entropy(B)=-\left(\frac{y}{z}\right)\times \log_{2}\left(\frac{y}{z}\right)-\left(\frac{z-y}{z}\right)\times \log_{2}\left(\frac{z-y}{z}\right)$$

with *p *+ *q *= *N*, *r *+ *z *= *N and x *+ *y *= *p*

*Information Gain *= *I (p, q) *- *Entropy (i)*

The variable that produces the largest information gain is selected for splitting since it maximizes the proportions of positive observations in the branches created by splitting the data according to its categories. Continuous variables are treated as categorical where the split in the range of values that produces the largest information gain is selected as cut-off point.

The stopping rule used was arbitrarily defined as a minimum of 200 observations per terminal node. The evaluation of the test error of each tree was conducted using a 10-fold cross-validation, which is considered appropriate for datasets containing at least 1,000 cases [37]. This process involves splitting the dataset into 10 mutually exclusive random segments, using 9 of them jointly for building the tree (training data) and the 10th as a test dataset to measure the error rate of the training sample (test data). This process is repeated until all the 10 segments of the dataset have been used once each as test sets. The 10 error estimates are averaged to yield an overall error estimate.

Sensitivity, specificity, predicted values and AUC of test data are reported for each tree. In order to maximize the detection of positive holdings, i.e. increase the sensitivity; the algorithm was forced to predict the outcome with the least expected misclassification cost using the meta-learner cost-sensitive classifier implemented in WEKA [36]. This cost was set for four different false negative/false positive ratios 1:1, 5:1, 10:1 and 20:1.

The movement data variables, all of which are on a continuous scale, were analysed using exploratory factor analysis to reduce this set of twelve variables to a smaller number of latent uncorrelated factors. Preliminary assessment of the suitability of the technique was done by checking the correlation of the 12 variables using pairwise correlation coefficients and by the Kaiser-Meyer-Olkin (KMO) criterion. The KMO criterion should be 0.6 or higher indicating that there is sufficient correlation between the variables to justify the use of factor analysis [38]. A provisional solution was extracted using principal components (PCF) and only factors with eigenvalues in the correlation matrix of the twelve variables greater than unity were considered [39]. Then the factor solution was optimized using an orthogonal varimax rotation to maximize the variances for each of the selected factors. The interpretation of the factors was based on the strength of the loadings of the variables on each of the selected factors and heuristically, by considering the biological interpretation of the resulting factors. The individual scores for the selected factors were tested for their association with the outcome of interest using multivariable logistic regression. All statistical analyses were performed using STATA 9.2 (Stata^® ^Corporation 2005).

The three data mining techniques applied, namely, logistic regression, classification trees and factor analysis, were used to investigate the structure of the dataset. Logistic regression determines the linear relationship between a set predictors and a dichotomous outcome, usually measured by the odds ratios as index of likelihood. It requires complete data, is sensitive to outliers and deal with interactions manually. The high co-linearity amongst variables may lead to large standard errors and imprecise estimation of the regression coefficients [40]. The classification trees are able to detect non-linear dependencies, handle interactions automatically, are less affected by missing values than logistic regression and use their classification ability as the only measure of the relationship of a set of predictors and the outcome [41]. The classification tree algorithm C 4.5 is able to handle missing values by using a complex procedure. A probability is assigned to all possible values/categories of a variable according to their frequency. Then a fraction of the observations with missing values in the variable equal to the probability for each value/category is distributed randomly down its correspondent branch and the information gain ratio computed to continue the split of the data or not [42].

Factor analysis describes a set of variables in terms of a smaller number of uncorrelated indices or factors in order to get a better understanding of the relationship between them [39]. The use of factor analysis allowed us to include the key features of movement patterns re-expressed as factor variables in the multivariable analyses.

Logistic regression and classification trees can be applied in a complementary fashion. The variables included in the final logistic regression model which have the largest odds ratios also appear at the top of the classification trees. When working with large datasets including hundreds of multi-categorical variables, classification trees can be used to perform a much less computationally demanding pre-selection of variables which can be then included in logistic regression analysis or any other. They can also provide indicative cut-off points for the categorization of numeric variables [25,43]. The elimination of non-significant variables in regression analysis based on backward variable selection is similar to the pruning of the tree that contains the maximum number of terminal nodes. In both cases the aim is to reach a balance between overfitting the data by building a model that completely reflects the training data and a more parsimonious one with less classification ability but that is easier to interpret.

## Authors' contributions

AO–P designed the study, conducted the analysis and wrote the manuscript. DUP supervised the study and corrected the manuscript. All authors read and approved the final manuscript.
